# Opposing Actions of Adrenocorticotropic Hormone and Glucocorticoids on UCP1-Mediated Respiration in Brown Adipocytes

**DOI:** 10.3389/fphys.2018.01931

**Published:** 2019-01-17

**Authors:** Katharina Schnabl, Julia Westermeier, Yongguo Li, Martin Klingenspor

**Affiliations:** ^1^Chair for Molecular Nutritional Medicine, TUM School of Life Sciences Weihenstephan, Technical University of Munich, Freising, Germany; ^2^EKFZ – Else Kröner-Fresenius Zentrum for Nutritional Medicine, Technical University of Munich, Freising, Germany; ^3^ZIEL – Institute for Food & Health, Technical University of Munich, Freising, Germany

**Keywords:** glucocorticoids (GC), brown adipose tissue, non-adrenergic activation, non-shivering thermogenesis, uncoupling protein 1, adrenocorticotropic hormone, obesity

## Abstract

Brown fat is a potential target in the treatment of metabolic disorders as recruitment and activation of this thermogenic organ increases energy expenditure and promotes satiation. A large variety of G-protein coupled receptors, known as classical drug targets in pharmacotherapy, is expressed in brown adipocytes. In the present study, we analyzed transcriptome data for the expression of these receptors to identify potential pathways for the recruitment and activation of thermogenic capacity in brown fat. Our analysis revealed 12 G_s_-coupled receptors abundantly expressed in murine brown fat. We screened ligands for these receptors in brown adipocytes for their ability to stimulate UCP1-mediated respiration and *Ucp1* gene expression. Adrenocorticotropic hormone (ACTH), a ligand for the melanocortin 2 receptor (MC2R), turned out to be the most potent activator of UCP1 whereas its capability to stimulate *Ucp1* gene expression was comparably low. Adrenocorticotropic hormone is the glandotropic hormone of the endocrine hypothalamus–pituitary–adrenal-axis stimulating the release of glucocorticoids in response to stress. In primary brown adipocytes ACTH acutely increased the cellular respiration rate similar to isoproterenol, a β-adrenergic receptor agonist. The effect of ACTH on brown adipocyte respiration was mediated via the MC2R as confirmed by using an antagonist. Inhibitor-based studies revealed that ACTH-induced respiration was dependent on protein kinase A and lipolysis, compatible with a rise of intracellular cAMP in response to ACTH. Furthermore, it is dependent on UCP1, as cells from UCP1-knockout mice did not respond. Taken together, ACTH is a non-adrenergic activator of murine brown adipocytes, initiating the canonical adenylyl cyclase–cAMP–protein kinase A-lipolysis-UCP1 pathway, and thus a potential target for the recruitment and activation of thermogenic capacity. Based on these findings in primary cell culture, the physiological significance might be that cold-induced ACTH in concert with norepinephrine released from sympathetic nerves contributes to BAT thermogenesis. Notably, dexamethasone attenuated isoproterenol-induced respiration. This effect increased gradually with the duration of pretreatment. *In vivo*, glucocorticoid release triggered by ACTH might oppose beta-adrenergic stimulation of metabolic fuel combustion in BAT and limit stress-induced hyperthermia.

## Introduction

We are facing a worldwide epidemic of obesity, a major risk factor in the development of non-communicable diseases such as diabetes mellitus and arteriosclerosis. In 2016, 1.9 billion adults aged 18 and over were overweight, of which 650 million were obese, representing an almost threefold increase in obesity prevalence since 1975 (WHO, 2018). Obesity is the state of excessive white adipose tissue (WAT) accumulation caused by prolonged positive energy balance. In mammals there is a second type of adipose tissue, brown adipose tissue (BAT), which in contrast to WAT generates heat in response to cold exposure and food consumption (Rosen and Spiegelman, 2006; Vosselman et al., 2013; U Din et al., 2018). It dissipates the chemical energy of macro-nutrients by uncoupling oxygen consumption from ATP synthesis in mitochondria (Klingenspor, 2003). This mechanism, known as non-shivering thermogenesis, is dependent on the presence of mitochondrial uncoupling protein 1 (UCP1), which is a unique feature of brown adipocytes. The activation of BAT increases energy expenditure and opposes positive energy balance. In addition to the activation of UCP1, it is worth noting that cold exposure also induces the thermogenic gene expression program and thereby recruits thermogenic capacity in brown fat (Cannon and Nedergaard, 2004). Furthermore, brown-like adipocytes, also known as inducible brown fat cells, beige (Ishibashi and Seale, 2010) or “brite” (brown-in-white) (Petrovic et al., 2010) adipocytes, can be found interspersed in WAT (Li et al., 2014a). Similar to brown adipocytes in classical brown fat depots brite adipocyte abundance can be increased by adrenergic stimulation (Galmozzi et al., 2014) and cold exposure (Maurer et al., 2015) in a process coined browning of WAT. Besides activation and recruitment of BAT, the browning of WAT displays therapeutic potential with regard to the development of new obesity treatment strategies.

For a long time, the occurrence of metabolically active BAT was believed to be restricted to hibernators, small mammals and human newborns. Adult humans, however, also have metabolically active BAT, as demonstrated by the detection of cold-induced uptake of tracers for glucose, fatty acids and acetate with positron emission tomography (Cypess et al., 2009, 2015; van Marken Lichtenbelt et al., 2009; Virtanen et al., 2009; Ouellet et al., 2011). Furthermore, cold-induced BAT activity is strongly reduced in obese (Saito et al., 2009) and diabetic patients and can be recovered by cold acclimation (van Marken Lichtenbelt et al., 2009).

On this background BAT displays a focal point of current research as it harbors a remarkable capacity to evoke energy expenditure through UCP1 dependent energy dissipation. The recruitment and activation of BAT and appears as an attractive and potentially effective strategy for the prevention and treatment of obesity (Tseng et al., 2010). Importantly, in the attempt to increase energy expenditure, the recruitment of more brown adipocytes with higher UCP1 expression and higher respiration capacity is required, but not sufficient. UCP1 is not constitutively active, but rather must be activated to dissipate mitochondrial proton-motive force as heat. This activation of UCP1 is inevitable to increase carbohydrate and lipid oxidation. Beyond boosting energy expenditure, we recently demonstrated that meal-induced activation of BAT thermogenesis also induces satiation which might also be applicable to promote negative energy balance (Li et al., 2018).

As sympathomimetic drugs exhibit unwanted detrimental cardiovascular effects, their application as BAT-stimulating agents is considered problematic (Cypess et al., 2015). Thus, the identification of druggable non-adrenergic regulators of BAT is one step toward the modulation of the heating organ as a regulator of energy expenditure and body fat in humans.

Besides the β-adrenergic receptors mature brown adipocytes express a variety of around 230 other G protein-coupled receptors (GPCRs) (Klepac et al., 2016) which are responsible for transferring extracellular signals to the cytosol. GPCRs group in a large family of seven transmembrane proteins (Lefkowitz, 2007; Kobilka, 2011) that regulate important biological processes in diverse tissues including adipose tissues (Wettschureck and Offermanns, 2005; Latek et al., 2012). Approximately 30% of all approved drugs target GPCRs, illustrating their importance in disease and therapeutics (Hauser et al., 2017; Santos et al., 2017). These receptors are coupled to heterotrimeric G proteins which are composed of α, β, and γ subunits. Ligand binding and thus activation of GPCRs leads to the dissociation of Gα from the Gβγ dimer, allowing the binding and regulation of signaling effectors. The downstream signaling of GPCRs is in part determined by their G protein coupling (Neves et al., 2002). There are four main sub-classes of Gα proteins: G_s_, G_i_, G_q_ and G12/13. Activation of G_s_ and G_i_ leads to the stimulation or inhibition the second messenger cyclic adenosine monophosphate (cAMP), respectively, while G_q_ activates phospholipase C und thus, to an increase of inositol triphosphate. G_12/13_ activates the small GTPase Rho, a pathway also known to be modulated by G_q_ family proteins (Buhl et al., 1995; Wang et al., 2013). Due to cAMP-PKA activating properties the analysis of BAT GPCRs has mainly focused on G_s_-coupled receptors [for example, β-adrenergic (Cannon and Nedergaard, 2004) and adenosine receptors (Gnad et al., 2014)] as they have the potency to activate UCP1-dependent thermogenesis. Cold-exposure induced release of norepinephrine from sympathetic nerves in BAT activates canonical adenylyl cyclase – cAMP – protein kinase A (PKA) signaling via β-adrenergic receptors. This pathway stimulates lipolysis and activation of UCP1 and therefore induces non-shivering thermogenesis (Klingenspor et al., 2017). Lipolysis plays a crucial role and is an essential requirement for UCP1 activation. Indeed, pharmacological inhibition of ATGL and HSL, the two lipases which catalyze the first two steps in the hydrolysis of triglycerides, completely diminishes adrenergic stimulation of thermogenesis (Li et al., 2014b).

The aim of the present study was to investigate a selection of non-adrenergic G_s_-coupled GPCRs in the light of their ability to activate and recruit UCP1-mediated thermogenesis in brown adipocytes.

## Materials and Methods

### Materials

Murine ACTH_(1-39)_ trifluoroacetate salt was purchased from Bachem (H-4998), ACTH_(4-10)_ was ordered from Abcam (ab142255), and the synthetic ACTH_(4-10)_ analog was synthesized and obtained from JPT Peptide Technologies GmbH. H89 was purchased from Tocris. Atglistatin and Hi 76-0079 were a kind gift from Prof. Robert Zimmermann. All other chemicals were ordered from Sigma unless otherwise specified. MC2R antagonist GPS1573 was purchased from Abbiotech (Bouw et al., 2014).

### Animals and Primary Cell Culture

All mice were bred at the specific-pathogen free animal facility of the Technical University of Munich registered at the local authorities according to §11 of the German Animal Welfare Act (Az32-568, 01/22/2015). In the present study, mice were killed for the dissection of tissues in deep CO_2_ anesthesia as approved by the ethics committee of the state supervisory authority (Government of Upper Bavaria). They had *ad libitum* access to food and water and were maintained at 22 ± 1°C and 50–60% relative humidity in a 12 h:12 h light:dark cycle. Male 129S6/SvEvTac, 129S1/SvEvTac mice (UCP1^-/-^ mice and wild-type littermates UCP1^+/+^) and heterozygous C57BL/6N Ucp1 dual-reporter gene mice (C57BL/6NTac-Ucp1tm3588 ^(Luciferase-T2A-iRFP-T2A-Ucp1)Arte^ named here as Ucp1^+/ki^) aged 5–6 weeks, were used to prepare primary cultures of brown and white adipocytes. Latter simultaneously express firefly luciferase and near-infrared fluorescent protein 713 (iRFP713). The *Luciferase-T2A-iRFP713-T2A* sequence was introduced into the 5′-untranslated region of the endogenous *Ucp1* gene (Wang et al., 2018). Interscapular brown and inguinal WATs were dissected and digested with collagenase as described previously (Li et al., 2014a). Stromal vascular fraction cells were seeded, grown to confluency and differentiated into mature adipocytes following a standard protocol. Adipocyte differentiation was induced for 48 h with 5 μg/ml insulin, 1 nM 3,3′,5-triiodo-l-thyronine (T3), 125 μM indomethacin, 500 μM isobutylmethylxanthine (IBMX) and 1 μM dexamethasone in adipocyte culture media (DMEM supplemented with 10% heat-inactivated FBS, penicillin/streptomycin). Cells were then maintained in adipocyte culture media supplemented with 5 μg/ml insulin and 1 nM T3 for 6 days with media changes every 2 days. Assays were performed on day 7 of differentiation.

### Luciferase Assay

After overnight stimulation of primary brown adipocytes of Ucp1^ki/ki^ mice luciferase activity was assayed using a commercial kit system (Luciferase Assay System Freezer Pack E4030, Promega GmbH). Primary cells were lysed in 1x reporter lysis buffer by shaking for 20 min at room temperature. 10 μl lysate was mixed with 50 μl luciferase assay substrate solution, and the mixture was measured by FB12 in a luminometer (Single Tube Luminometer, Titertek-Berthold GmbH). Bioluminescence readouts were normalized to total protein content.

### Respiration Assays

Oxygen consumption of primary brown adipocytes was measured at 37°C using microplate-based respirometry (XF96 extracellular flux analyzer, Seahorse Bioscience) as described previously (Li et al., 2014b) following the subsequent protocol at day 7 of differentiation. Prior to the respiration measurement, primary cells were washed with warmed, unbuffered assay medium (DMEM basal medium supplemented with 25 mM glucose, 31 mM NaCl, 2 mM GlutaMax and 15 mg/l phenol red, pH 7.4) (basal assay medium). Subsequent to the medium replacement with basal assay medium containing 1–2% essentially fatty acid free bovine serum albumin (BSA), cells were incubated at 37°C in a CO_2_-free incubator for 1 h. Assay reagents were loaded into the drug injections ports of the sensor cartridges at 10X in basal assay medium (no BSA). After assessment of basal oxygen consumption in untreated cells oligomycin (5 μM) was injected to inhibit coupled respiration and to determine basal leak respiration. Next, effector was added to investigate UCP1-dependant uncoupled respiration. By the addition of FCCP (1 μM) maximal respiratory capacity was determined. Lastly, non-mitochondrial oxygen consumption was assessed by blocking mitochondrial respiration with antimycin A (5 μM). For some experiments, cells were pretreated for 1 h with 50 μM H89 (PKA inhibitor), 1–100 μM propranolol (β-adrenergic receptor antagonist), 40 μM Atglistatin (ATGL inhibitor) and 40 μM Hi76-0079 (HSL inhibitor) 1 h prior to the measurement. Oxygen consumption rates were automatically calculated by the Seahorse XF-96 software. Each experiment was repeated at least three times with similar results and five to eight replicate wells for every condition in each independent experiment. Results are predominately expressed as stimulated respiration which is calculated as fold increase of basal leak.

### Gene Expression Analysis (qRT-PCR)

Total RNA was isolated using Trisure (Bioline) and purified with SV total RNA Isolation System (Promega). Reverse transcriptase reactions were performed using SensiFAST cDNA Synthesis Kit (Bioline). Quantitative real-time PCR (qRT-PCR) was performed with SYBR green fluorescent dye in 384-well format using LightCycler 480 (Roche). General Transcription Factor IIB (Gtf2b) served as an internal control. To be able to calculate relative gene expression levels of samples, standard reactions containing serial diluted pooled cDNA of all samples (Pure, 1:2, 1:4, 1:8, 1:16, 1:32 and 1:64) as a template were used to establish a standard curve. The RNA abundance of *Ucp1* gene was normalized to the housekeeping gene Gtf2b. The following primers were used:

Ucp1 F: 5′-GTACACCAAGGAAGGACCGA-3′, R: 5′-TTTATTCGTGGTCTCCCAGC-3′;
Gtf2b F: 5′-TGGAGATTTGTCCACCATGA-3′, R: 5′-GAATTGCCAAACTCATCAAAACT-3′.

### Western Blot Analysis

Primary brown adipocytes were lysed in RIPA buffer for western blot analysis. 30 μg of total lysates were separated by SDS-PAGE (12.5% gels), transferred to Odyssey^®^ nitrocellulose membrane (Millipore), and probed with anti-UCP1 (1:10,000; ab10983, Abcam), and anti-Actin (1:10,000; MAB1501, Millipore). Secondary antibodies conjugated to IRDye^TM^ 680 or IRDye^TM^ 800 (Licor Biosciences) were incubated at a dilution of 1:20,000. Fluorescent images were captured by Odyssey infrared imaging system (Licor Biosciences).

### Quantification of Cellular cAMP

Changes in cytosolic cAMP concentrations were determined in primary brown adipocytes in response to ACTH- or isoproterenol stimulation using cAMP-Glo Assay (Promega) following the manufacturer’s instructions.

### Statistical Analysis

Significant differences for single comparisons were assessed by two-tailed Student’s *t*-test. Analysis of variance (ANOVA) with Tukey’s *post hoc* tests were used for multiple comparisons (GraphPad Prism 6.0 software). *P*-values < 0.05 were considered a statistically significant difference. All data are presented as mean ± SD.

## Results

### G_s_-Coupled GPCRs Abundantly Expressed in Brown Adipose Tissue

**FIGURE 1:**
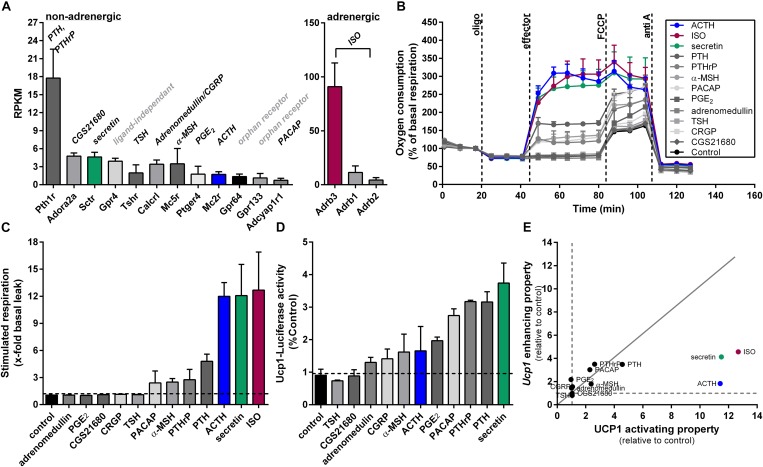
Screening approach of G_s_PCR ligands expressed in brown adipose tissue concerning their ability to activate and recruit Ucp1. **(A)** Gene expression of G_s_-protein coupled receptors (G_s_PCRs) obtained from RNA sequencing of murine brown adipose tissue. Data are shown as RPKM (reads per kilobase per million mapped reads). The respective ligands of the receptors are shown in italics above the bars. G_s_PCRs with a lower expression have been neglected for the analysis (*n* = 4). **(B)** Microplate-based respirometry of primary brown adipocytes following the subsequent protocol. After assessment of basal oxygen consumption oligo (oligomycin) was injected to determine basal leak respiration. Next, compound to be tested was added to investigate uncoupling protein 1-dependant respiration. By the addition of FCCP maximal leak respiration was determined. Lastly, non-mitochondrial oxygen consumption was assessed by injecting anti A. Shown is a time course of oxygen consumption in percentage of basal respiration of primary brown adipocytes (129S6 mice) in response to different stimuli (effectors). **(C)** Stimulated respiration of primary brown adipocytes as fold increase of basal leak respiration (*n* = 5). **(D)** Overnight stimulation of primary brown adipocytes from Ucp1^ki/ki^ mice expressing the firefly luciferase driven by the gene regulatory elements of endogenous *Ucp1* gene. Measurement of luciferase activity as surrogate for Ucp1 expression (*n* = 3). **(E)** Plot of Ucp1 enhancing properties against UCP1 activating properties of screened ligands relative to control. Dashed lines indicate control levels (=1), continuous gray line indicates line of equality. Data are presented as means ± SD. ***Pth1r***, parathyroid hormone 1 receptor; ***Adora2a***, adenosine receptor; ***Sctr***, secretin receptor; ***Gpr4***, GPCR4; ***Tshr***, thyroid stimulating hormone receptor; ***Calcrl***, calcitonin receptor-like receptor; ***Mc5r***, melanocortin receptor 5, ***Ptger4***, prostaglandin E receptor 4; ***Mc2r***, melanocortin receptor 2; ***Gpr64***, GPCR 64; ***Gpr133***, GPCR 133; ***Adcyap1r1***, adenylate cyclase activating polypeptide 1 receptor; **TSH**, thyroid stimulating hormone; **CGS21680**, adenosine A2A receptor agonist; **CGRP**, calcitonin gene-related peptide; **α-MSH**, melanocyte-stimulating hormone; **ACTH**, adrenocorticotropic hormone; **PGE_2_**, prostaglandin E2; **PTHrP**, parathyroid hormone related peptide; **PTH**, parathyroid hormone; **ISO**, isoproterenol; **oligo**, oligomycin; **FCCP**, carbonyl cyanide 4-(trifluoromethoxy) phenylhydrazone; **anti A**, antimycin A.

In our search for non-adrenergic targets to activate and recruit UCP1 in BAT, we identified 12 G_s_-protein coupled receptors (G_s_PCRs) expressed in the interscapular BAT of mice (C57BL/6J, 12 weeks old, room temperature, chow diet) at different abundances (threshold was set at RPKM > 1) based on our recently published RNA-seq data (GEO: GSE119452). Among these G_s_PCRs, parathyroid hormone receptor (*Pthr*) showed the highest abundance, whereas adenylate cyclase activating polypeptide 1 receptor concluded with the lowest expression (Figure 1A). GPCR64 and GPCR133 turned out to be adhesion receptors and GPCR4 is reported to carry out ligand independent signaling mediated through proton-sensing mechanisms (Ludwig et al., 2003; Bohnekamp and Schoneberg, 2011). We therefore excluded these three receptors for further investigations. In comparison to non-adrenergic receptors, the genes encoding for the β-adrenergic receptors ADRB1/2/3 showed most abundant expression in murine BAT, with *Adrb3* standing out with the highest expression. We used Isoproterenol, a non-selective β-adrenoreceptor agonist, to activate β-adrenergic signaling in brown adipocytes. For all other non-adrenergic receptors, the corresponding ligands were selected to test their capability to activate and recruit UCP1. In case of PTHR and calcitonin gene-related peptide receptor (CALCRL), two established ligands were chosen (Chang et al., 2004; Dean et al., 2006).

### Reporter-Assays and Cellular Respirometry Screening of G_s_-Coupled GPCR Ligands for UCP1 Activity and Regulation

In our first screen, we tested whether these G_s_PCR ligands can activate oxygen consumption acutely in brown adipocytes. UCP1-mediated uncoupled respiration was quantified in cultured adherent intact primary brown adipocytes, according to a protocol established in our lab (Li et al., 2014b). After recording basal respiration and the fraction of coupled and uncoupled respiration, UCP1-dependent respiration was measured. Finally, maximal respiratory capacity and non-mitochondrial oxygen consumption were assessed by adding the uncoupling agent carbonyl cyanide 4-(trifluoromethoxy) phenylhydrazone (FCCP) and antimycin A was added to block the electron transport chain, respectively. All respiration assays were performed in the presence of BSA to buffer free fatty acid (FFA) levels in the respiration medium. For ACTH and secretin, we observed the most prominent induction of UCP1-dependant uncoupled respiration. The potency of these two peptides was comparable to ISO, the β-adrenergic receptor agonist. Both PTH and its related peptide both stimulated UCP1-dependant respiration, although the effect was modest compared to ISO. The weakest effects on cellular respiration were observed for PACAP and α-MSH. All other ligands failed to increase basal leak respiration (Figures 1B,C).

We then measured FLUC activity in primary brown adipocytes from Ucp1^ki/ki^ reporter gene mice to determine the effect of the G_s_PCR ligands on *Ucp1* gene expression in response to overnight stimulation. As the expression of *Fluc* in these cells is driven by *Ucp1*promoter, FLUC activity served as a surrogate for *Ucp1* expression. We found highest FLUC activities in response to secretin, parathyroid hormone (PTH), parathyroid hormone related peptide (PTHrP), and pituitary adenylate cyclase-activating peptide (PACAP) (Figure 1D). Prostaglandin E2 (PGE2), adrenocorticotropic hormone (ACTH) and α-melanocyte-stimulating hormone (α-MSH) showed a moderate rise in FLUC activity. The two ligands for calcitonin receptor-like receptor (CALCRL), calcitonin gene-related peptide (CGRP), and adrenomedullin only had minor effects, whereas thyroid hormone and the adenosine 2A receptor agonist CGS21680 had no effect on FLUC (Figure 1D). Comparing the effect sizes for all ligands on the ability to activate respiration and to recruit *Ucp1* expression, ACTH and secretin were most potent to acutely activate respiration. Notably, although secretin, PTH and PTHrP showed comparable strong induction of FLUC activity, PTH and PTHrP were much less potent as secretin in the acute activation assay. Moreover, ACTH matched secretin in the acute activation assay, but was much less potent to induce FLUC (Figure 1E). Based on the pronounced and relatively preferential stimulatory effect of ACTH on UCP1-mediated respiration we chose the ACTH to further investigate its thermogenic properties.

### ACTH Activates Respiration via the Canonical cAMP-PKA-Lipolysis Pathway

The hypothalamic–pituitary–adrenal (HPA) axis plays a critical role in maintaining homeostasis and in mounting appropriate responses to stress. Key components of the stress response are aimed at providing adequate amounts of glucocorticoids which exert pleiotropic effects on energy supply, fuel metabolism, immunity and cardiovascular function. The melanocortin ACTH is the glandotropic hormone of the HPA which stimulates synthesis and release of glucocorticoids by the cortex of the adrenal gland. The 39 amino acid peptide hormone is synthesized within the anterior pituitary by corticotropic cells as a much larger, 241-amino-acid precursor known as pro-opiomelanocortin (POMC) in response to tonic control from the hypothalamus by corticotrophin-releasing hormone (CRH). ACTH is able to activate all five G_s_-protein coupled melanocortin receptors, but at physiological circulating plasma levels, the sensitivity of all receptors, except MC2R, is so low that they are not activated. In rodent adipocytes, ACTH binds to the MC2R, which stimulates lipolysis via G_s_-coupled cAMP-PKA mediated phosphorylation of HSL (Cho et al., 2005).

**FIGURE 2:**
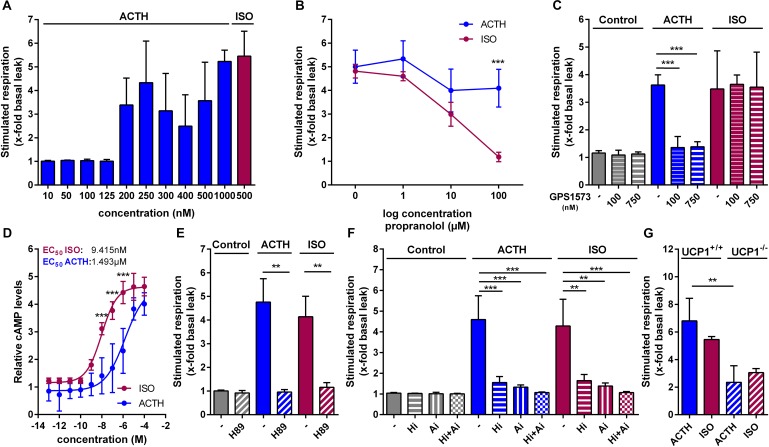
ACTH activates UCP1 via the canonical cAMP-PKA-lipolysis pathway. Oxygen consumption of primary brown adipocytes was assessed using microplate-based respirometry. First, basal respiration was determined and then, oligomycin (oligo) was added, which inhibits adenosine triphosphate (ATP) synthase resulting in basal leak respiration. UCP1 mediated uncoupled respiration was determined after injection of isoproterenol (ISO, 0.5 μM) as a positive control or adrenocorticotropic hormone (ACTH, 1 μM). Next, carbonyl cyanide 4-(trifluoromethoxy) phenylhydrazone (FCCP), an uncoupler that allows assessment of maximal respiratory capacity, was added. Finally, antimycin A (anti A) was added in order to determine non-mitochondrial respiration. **(A)** Dose–response experiment revealing most robust effects at a ACTH concentration of 1 μM assessed as stimulated respiration as fold increase of basal leak respiration. **(B)** ACTH- and ISO-stimulated respiration after 1 h pretreatment with different concentrations of propranolol, a non-selective blocker of adrenergic β-receptors (*n* = 4). **(C)** Effects of different concentrations of GPS1573 (100 nM and 750 nM), a MC2R antagonist, on ACTH- and ISO-stimulated UCP1-mediated uncoupled respiration. **(D)** Cytosolic cAMP abundance after stimulation with increasing ACTH and ISO concentrations for 30 min (*n* = 3). **(E)** Fold increase of basal leak respiration after stimulation with ISO, ACTH and vehicle (control, assay medium) with or without protein kinase A inhibitor H89 (50 μM). Inhibitor was injected together with oligo prior to addition of stimulators (*n* = 4). **(F)** Respiration stimulated by ISO, ACTH or vehicle as fold increase of basal leak after 1 h pre-treatment with lipolysis inhibitors Ai (Atglistatin, ATGL-inhibitor, 40 μM) and Hi (Hi76-0079, HSL-inhibitor, 40 μM) (*n* = 4). **(G)** Stimulated respiration of primary brown adipocytes from UCP1^+/+^ and UCP1^-/-^ mice (*n* = 3). Data are presented as means ± SD. **(B,D,G)** were analyzed by two-way ANOVA (Tukey’s test). **(C**,**E,F)** were analyzed by unpaired *t*-test. **(D)** EC_50_ was determined by non-linear regression analysis. ^∗^*p* < 0.05, ^∗∗^*p* < 0.01, ^∗∗∗^*p* < 0.001.

Since ACTH was previously reported to stimulate uncoupled respiration in the immortalized murine white fat cell line T37i (van den Beukel et al., 2014), we next investigated UCP1-mediated respiration and its respective signaling pathway in primary brown adipocytes. In a dose–response experiment we found that 200 nM ACTH stimulated respiration, defined as fold increase of respiration over basal leak respiration, with the most robust effect at 1 μM ACTH (Figure 2A). This effect of ACTH on brown adipocyte respiration was independent of adrenergic receptors, since pre-treatment of the cells with propranolol, a non-selective β-adrenergic receptor antagonist, did not attenuate ACTH stimulated respiration while blocking the effect of ISO in a dose-dependent manner (Figure 2B). The effect of ACTH, however, depends on MC2R, as pretreatment with the MC2R antagonist GPS1573 (100 nM and 750 nM) blunted the effects of ACTH on oxygen consumption (Figure 2C). ACTH stimulation of primary brown adipocytes resulted in a dose-dependent increase of cytosolic cAMP levels. Compared to ISO, however, EC_50_ of ACTH was approximately 165-fold higher (9.415 nM vs. 1.493 μM) (Figure 2D). Pre-treatment of cells with H89, a selective inhibitor of PKA (Figure 2E), or inhibitors targeting the two essential lipases involved in lipolysis (ATGL, adipose triglyceride lipase; HSL, hormone-sensitive lipase), completely blocked the effect on respiration of ACTH (Figure 2F). The stimulating effect of ACTH depended on UCP1 as it was attenuated in primary brown adipocytes of UCP1^-/-^ compared to UCP1^+/+^ (Figure 2G). As the ACTH-induced stimulated respiration was dependent on UCP1, we consider the effect of the ACTH on brown adipocytes as thermogenic.

Taken together, ACTH activates UCP1-dependent respiration in primary brown adipocytes via the canonical cAMP-PKA-lipolysis pathway.

### ACTH Increases *Ucp1* Expression in Primary Brown and White Adipocytes

**FIGURE 3:**
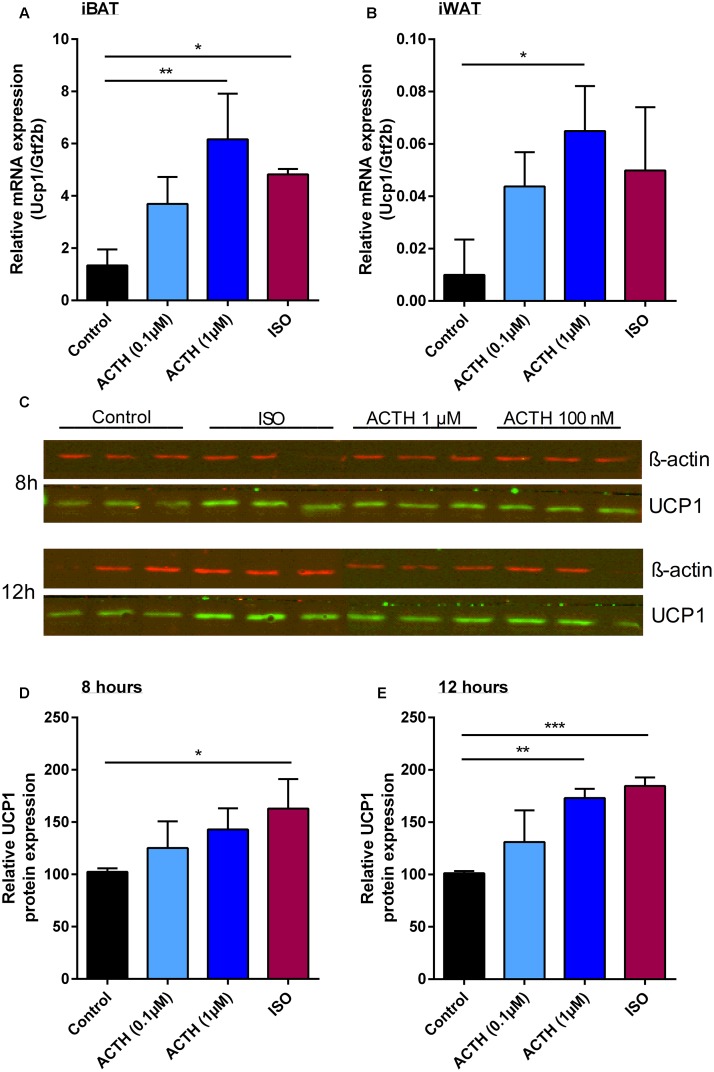
ACTH increases Ucp1 expression in primary brown and white adipocytes. Relative expression of uncoupling protein 1 (*Ucp1*) normalized to transcription factor *Gtf2b* in primary adipocytes from both **(A)** interscapular brown (iBAT) and **(B)** inguinal white adipose tissue (iWAT) in response to isoproterenol (ISO) or adrenocorticotropic hormone (ACTH). **(C)** Western blot analysis of UCP1 and β-actin in primary inguinal adipocytes after stimulation with ISO or ACTH for 8 and 12 h. Relative UCP1 protein expression normalized to β-actin in primary inguinal white adipocytes after treatment with differentiation medium (control), ACTH (0.1 μM), ACTH (1 μM) or ISO (0.5 μM) for **(D)** 8 or **(E)** 12 h. Data are presented as means ± SD (*n* = 4). **(A,B,D,E)** were analyzed by one-way ANOVA (Tukey’s test). ^∗^*p* < 0.05, ^∗∗^*p* < 0.01, ^∗∗∗^*p* < 0.001.

Next, we aimed to investigate the effect of ACTH on *Ucp1* gene expression in primary brown and white adipocytes. Indeed, treatment of differentiated brown and white adipocytes with two different doses of ACTH caused a dose-dependent increase in *Ucp1* mRNA levels (Figures 3A,B). The maximal induction of *Ucp1* mRNA achieved by 1 μM ACTH was comparable to the effect of ISO treatment (500 nM). Concomitant, UCP1 protein was also induced either by ACTH or ISO in primary brown adipocytes. This effect on gene expression was more pronounced after 12 h of stimulation compared to 8 h, and was dose-dependent. At a concentration of 1 μM, ACTH and ISO were equipotent in recruiting UCP1 protein in primary brown adipocytes (Figures 3C–E).

### A Synthetic ACTH Fragment Slightly Stimulates Respiration and Increases *Ucp1* mRNA Level in Primary Brown Adipocytes

**FIGURE 4:**
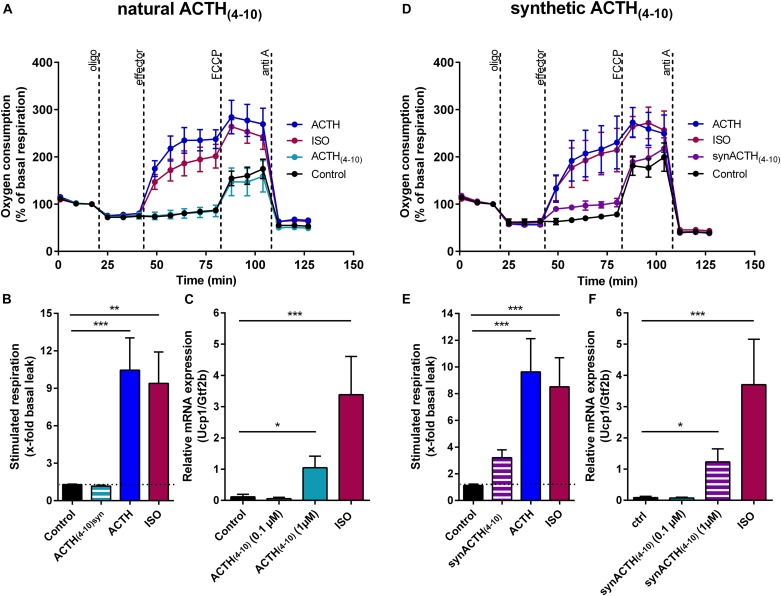
A synthetic ACTH fragment (heptapeptide) slightly stimulates respiration and increases Ucp1 mRNA level in primary brown adipocytes. **(A**–**C)** Heptapeptide core sequence of adrenocorticotropic hormone [ACTH_(4-10)_] and **(D**–**F)** a synthetic analog [ACTH_(4-10)_] having an amino acid exchange from Arg to Glu were analyzed with regard to their ability to recruit and activate uncoupling protein 1 (UCP1) in primary brown adipocytes. **(A**,**D)** First, basal respiration was assessed. Subsequently, adenosine triphosphate (ATP) inhibitor oligomycin (oligo) was added to define basal leak respiration. Next, UCP1-dependant uncoupled respiration was measured in response to 0.5 μM isoproterenol (ISO), ACTH or the heptapeptide fragments of ACTH (all 1 μM). The chemical uncoupler carbonyl cyanide 4-(trifluoromethoxy) phenylhydrazone (FCCP) was added to determine maximal respiratory capacity. Lastly, non-mitochondrial background was determined by addition of complex III inhibitor antimycin A (anti A). **(B**,**E)** Stimulated respiration was calculated as fold increase of basal leak respiration. **(C**,**F)** Relative expression of uncoupled protein 1 (*Ucp1*) normalized to transcription factor *Gtf2b* assessed by qPCR. Data are presented as means ± SD (*n* = 4). **(B,C,E,F)** were analyzed by one-way ANOVA (Tukey’s test). ^∗^*p* < 0.05, ^∗∗^*p* < 0.01, ^∗∗∗^*p* < 0.001.

We showed that both ACTH and α-MSH are capable in stimulating uncoupled respiration (Figures 1B,C). The heptapeptide sequence, Met-Glu-His-Phe-Arg-Trp-Gly, is common to all the adrenocorticotropic (ACTH), melanotropic (MSH), and lipotropic (LPH) hormones. Structure function studies on melanocortin peptides from the early 1970s using stimulation of lipolysis as a criterion for biological potency revealed that the heptapeptide core sequence exerts biological activity. An amino acid exchange from Glu to Arg in the ACTH core sequence resulted in a fourfold increased activity on the release of FFAs compared to the natural ACTH core sequence (Draper et al., 1973). As lipolysis is an essential prerequisite for the activation of UCP1, we included both the natural and the synthetic version of the ACTH fragment in our study. In primary brown adipocytes, the natural core sequence of ACTH [ACTH_(4-10)_] had no impact on respiration (Figures 4A,B), but significantly increased *Ucp1* mRNA expression, although not to the extent as seen for ISO (Figure 4C). The mutant ACTH fragment [synACTH_(4-10)_] showed limited potential to activate UCP1 as it mildly increased respiration (Figures 4D,E). The induction of *Ucp1* mRNA expression was comparable to that of the natural heptapeptide (Figure 4F). As it turned out, the synthetic ACTH fragment has potential to stimulate both thermogenic capacity and activity, and therefore is a potential non-adrenergic activator of BAT thermogenesis.

### Acute Glucocorticoid Treatment Attenuates β3-Adrenergic Signaling but Does Not Affect Thermogenic Effects of ACTH

**FIGURE 5:**
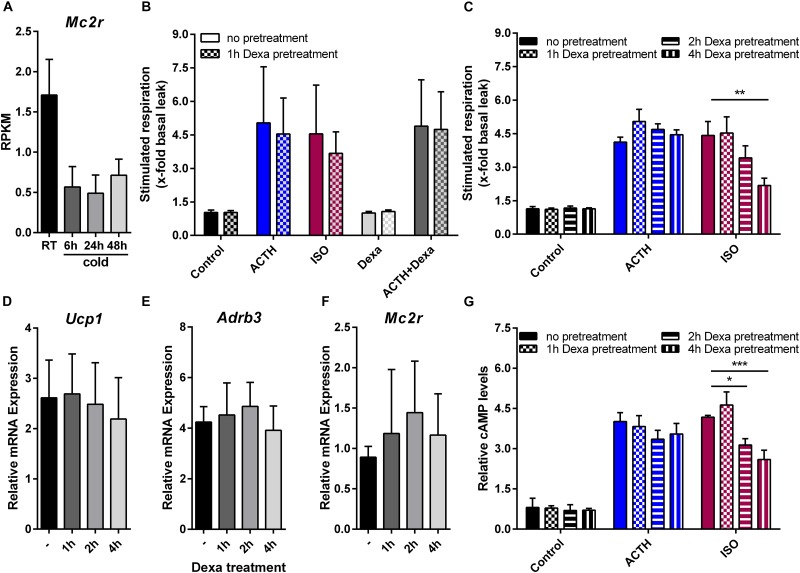
Acute glucocorticoid treatment attenuates β3 adrenergic signaling but does not affect non-adrenergic ACTH-signaling. **(A)** Gene expression of melanocortin 2 receptor (*Mc2r*) in response to varying ambient temperatures obtained from RNA sequencing of murine brown adipose tissue. Data are shown as RPKM (reads per kilobase per million mapped reads) **(B)** Primary brown adipocytes were either not pretreated or with dexamethason (Dexa) for 1 h prior to microplate-based respirometry. Subsequently, stimulated respiration as fold increase of basal respiration was determined in response to adrenocorticotropic hormone (ACTH), isoproterenol (ISO), Dexa or a combination of ACTH and Dexa. After either no or 1, 2 or 4 h of pretreatment with Dexa **(C)** ACTH- and ISO-stimulated respiration, relative expression of **(D)** uncoupling protein 1 (*Ucp1*), **(E)** β3-adrenergic receptor (*Adrb3*) and **(F)** *Mc2r* or **(G)** relative cellular cyclic aenosine monophosphate (cAMP) was assessed in primary brown adipocytes. Data are presented as means ± SD (*n* = 4). **(B**,**C**,**G)** were analyzed by two-way ANOVA (Tukey’s test). **(D–F)** were analyzed by one-way ANOVA. ^∗^*p* < 0.05, ^∗∗^*p* < 0.01, ^∗∗∗^*p* < 0.001.

To address the physiological role of ACTH in brown fat thermogenesis we analyzed the expression of its receptor in BAT of mice in response to cold exposure. Indeed, *Mc2r* is down-regulated in response to cold (Figure 5A) indicating that its contribution to cold-induced BAT thermogenesis might be rather minor. Nevertheless, subsequent to cold exposure plasma ACTH levels are significantly increased (van den Beukel et al., 2014). Basically, ACTH triggers the release of glucocorticoids from the cortex of the adrenal gland. Circulating glucocorticoids exert negative feedback control on the secretion of CRH and ACTH from the hypothalamus and the anterior pituitary. In addition, it is known that corticosterone reduces *Ucp1* mRNA and UCP1 protein in response to both adrenergic stimulation and ACTH treatment (Soumano et al., 2000; van den Beukel et al., 2014). Furthermore, glucocorticoids may exert rapid non-genomic effects mediated by a putative membrane-bound receptor as reported for the murine brain (Nahar et al., 2016; Shaqura et al., 2016). Therefore, we finally investigated the acute effect of treatment with the glucocorticoid dexamethasone (Dexa) on β-adrenergic and ACTH signaling. Therefore we pretreated primary brown adipocytes with 5 μM Dexa for 0–4 h and analyzed UCP1-dependent uncoupled respiration. One-hour pre-treatment as well as acute stimulation with Dexa had no effect on respiration (Figure 5B). With increasing exposure time Dexa attenuated ISO-stimulated respiration whereas ACTH-induced respiration was unaffected (Figure 5C). This was not a consequence of reduced or increased expression of the β3-adrenergic or the melanocortin 2 receptor (Figures 5D,E). Of note, although there was trend toward a decrease in *Ucp1* mRNA, a 4-h treatment with Dexa had no significant effect on *Ucp1* expression (Figure 5F). Our further analysis revealed that Dexa attenuated the ISO induced rise in cellular cAMP levels, whereas no such effect was observed for ACTH (Figure 5G). This implies that the inhibitory action of DEXA must occur upstream of cAMP.

## Discussion

Targeting brown fat to increase energy expenditure and promote negative energy balance has been a long sought strategy to prevent overweight and treat obesity (Tseng et al., 2010). In line with the hypothesis of thermoregulatory feeding (Himms-Hagen, 1995), our recent identification of an endocrine gut – secretin – brown fat – brain axis inhibiting food intake demonstrates that brown fat can also attenuate energy intake (Li et al., 2018). In addition to positive effects on energy balance, chronic activation of BAT leads to improved glucose tolerance and the release of batokines that beneficially regulate metabolism in rodent models (Bartelt et al., 2011; Hondares et al., 2011). Several novel molecular mediators for the recruitment of BAT and/or the browning of WAT have been found (Bartelt and Heeren, 2014), but only few direct activators of respiration in brown adipocytes were reported, so far (Braun et al., 2018). The latter mostly are adrenergic receptor agonists which exhibit unwanted systemic side effects (Cypess et al., 2012, 2015; Vosselman et al., 2012; Carey and Kingwell, 2013). Therefore, the present study was designed to reveal novel non-adrenergic activators of brown adipocytes. As the activation of the G_s_-coupled β_3_-adrenergic receptor leads to increased lipolytic activity by the canonical cAMP-PKA pathway providing FFAs essential for the activation of UCP1-mediated respiration, we screened for G_s_PCR expressed in mature murine adipocytes and assessed their capability to trigger the same signaling cascade. In our screen, we identified six peptidergic ligands of non-adrenergic G_s_PCR expressed in brown fat which acutely activated UCP1-mediated respiration in cultured brown adipocytes. Among these ligands, the glandotropic peptide hormone ACTH was one of the most potent activators of brown adipocyte respiration.

Some technical premises were of particular importance in our search for activators of UCP1-mediated respiration. Firstly, we tested the selected ligands for their acute effect on respiration. Secondly, by adding essentially fatty acid-free bovine serum into the respiration medium we prevented unspecific UCP1-independent uncoupling activity induced by uncontrolled high FFA levels (Li et al., 2014b). Thirdly, for ACTH we compared the effects in primary brown adipocytes from *wild-type* and *Ucp1* knockout mice. In previous studies identifying potential activators of brown adipocyte uncoupled respiration, either UCP1 specificity was uncertain due to the applied assay conditions, or the effects on respiration were due to mitochondrial biogenesis induced by long term stimulation with the putative activator.

Consistent with our recent report (Li et al., 2018), secretin was the strongest activator of UCP1-mediated uncoupled respiration, closely followed by ACTH. In comparison, the effects of PTH and PTHrP on respiration were rather minor, demonstrating some potential of these peptides to activate brown adipocytes, in addition to their demonstrated role in WAT browning (Kir et al., 2014). Furthermore, in line with the known pro-lipolytic activity of α-MSH and PACAP (Akesson et al., 2003; Lafontan, 2012), these peptides also showed low stimulation of brown adipocyte respiration.

Most peptides were about equipotent in stimulating respiration and gene expression, whereas secretin and ACTH showed a bias toward acute activation, thus resembling ISO (Figure 1E). Other than ACTH, the ACTH_(4-10)_ fragment stimulated *Ucp1* gene expression but failed to activate UCP1-dependant respiration (Figures 4B,C). This discrepancy is unexpected as activation and recruitment of BAT share a common signaling pathway that furcates at the level of PKA. Upon cAMP binding, activated PKA phosphorylates the lipid droplet coating proteins perilipin and HSL which are essential for lipolysis and acute activation of thermogenesis (Li et al., 2014b), as well as the transcription factor cAMP response element binding protein (CREB). In concert with activating transcription factor 2 (ATF-2) and PPAR coactivator 1α (PCG1α), both downstream of PKA, CREB induces *Ucp1* expression (Klingenspor et al., 2017). On this background, one would assume a fixed ratio for the increase in *Ucp1* expression and UCP1 activity in response to the G_s_-coupled activation of the canonical cAMP-PKA pathway. Alike isoproterenol, however, ACTH and secretin both prioritize acute activation of respiration over gene expression. This phenomenon, known as signaling bias, is due to a receptor’s ability to selectively engage specific subsets of downstream signaling modules. Bias may have multiple causes, including variation of ligand concentration and selective G-protein activation. Depending on concentration, one ligand can trigger variable activation of multiple cellular pathways (Kenakin, 1995), and GPCRs can interact with different G-proteins, arrestins and accessory proteins (Wootten et al., 2018). The secretin receptor for example couples to both G_s_- and G_q_-protein with opposing downstream signaling effects (Siu et al., 2006), and ACTH stimulates arrestin-dependent internalization of MC2R and MC2R accessory protein 1 (MRAP1) in a concentration dependent manner (Roy et al., 2011). The molecular mechanisms responsible for biased signaling of ACTH and secretin in brown adipocytes merit further investigation to identify signaling events that prioritize activation of UCP1.

In mice, ACTH increases thermogenic gene expression, hallmarked by *Ucp1*, in immortalized brown adipocytes (T37i) and in primary brown adipocytes, respectively (van den Beukel et al., 2014; Biswas, 2017). We here demonstrate acute activation of UCP1-dependent respiration in primary brown adipocytes by ACTH, as proven by the exclusion of unspecific uncoupling by fatty acids, the dose-dependent thermogenic response, and the knockout of UCP1. In combination, the pronounced acute stimulating effect on respiration and the induction of *Ucp1* gene expression qualify ACTH as a non-adrenergic effector candidate to boost activation of BAT thermogenesis *in vivo*. The discrepancy in the magnitude of ACTH effects on *Ucp1* expression assessed by either luciferase activity in our screening assay or *Ucp1* mRNA levels is probably due to strain differences of primary brown adipocytes, methodological differences and/or different duration of stimulation.

The melanocortins ACTH and α-MSH are known for their lipolytic action in rodent adipocytes, as first reported in 1958 (Lafontan, 2012; Braun et al., 2018). Cold exposure increased the plasma level of ACTH and in adipocytes it also progressively increased the responsiveness to this hormone (Rochon and Bukowiecki, 1990; van den Beukel et al., 2014). ACTH enhanced BAT function in obese rats (York and Al-Baker, 1984), and increased glucose transport and respiration in isolated brown adipocytes (Marette and Bukowiecki, 1990) via fatty acid activation of mitochondrial uncoupled respiration. In mouse strains with different stress reactivity, the physiological serum ACTH concentrations are variable, ranging from 12 to 27 pM at basal level (Ochedalski et al., 2001; Touma et al., 2008) and 133–246 pM after 15-min restraint stress (Heinzmann et al., 2010). In response to cold exposure (4°C for 24 h), serum ACTH levels rise up to 260 pM (van den Beukel et al., 2014). In cell culture, adipocytes respond to ACTH at concentrations ranging from 50 nM (T37i cells) to 100 nM (immortalized murine adipocytes) (Iwen et al., 2008; van den Beukel et al., 2014). We observed increased respiration rates at 200 nM, with most robust effects at 1 μM, with effective concentrations on *Ucp1* gene expression in a comparable range reported by others (Biswas, 2017). Thus, effective concentrations in cell culture are approximately 1,000-fold higher than the maximal physiological ACTH levels in response to restraint stress or cold exposure. To the best of our knowledge, no published studies are available that tested the effect of ACTH on brown adipocyte respiration at physiological doses. Treatment with a supraphysiological dose (15 μM ACTH, ∼50.000-fold above physiological levels), however, increased glucose uptake into BAT (van den Beukel et al., 2014). Pathophysiological chronic exposure to excessive concentrations of ACTH results in elevated glucocorticoid levels, as known for Cushing’s syndrome, with symptoms like visceral obesity, growth retardation, hirsutism, acne and hypertension (Bista and Beck, 2014; Lacroix et al., 2015), but no evidence for increased BAT activity. Further analysis is necessary to evaluate the thermogenic effect of physiological concentrations of ACTH *in vivo*. In a physiological context, it has to be taken into account that the sensitivity and affinity of the receptor *in vivo* might be different, or increased by certain stimuli such as cold.

In the attempt to reduce side effects as well as to improve efficacy, alternative MC2R ligands could be of advantage to develop strategies for tissue-specific MC2R activation or selective intracellular signaling. We therefore compared an ACTH heptapeptide representing the core sequence of melanocortins (Draper et al., 1975) with an synthetic analog of this ACTH fragment carrying a mutation in position 5 (Arg > Glu). Despite the high lipolytic activity reported for the synthetic analog ACTH_(4-10)syn_, it had only minor effects on brown adipocyte respiration, whereas the heptapeptide fragment was inactive. Thus, these alternative ligands are unsuitable to efficiently activate brown fat.

The glandotropic hormone ACTH induces the production and secretion of glucocorticoids (GCs) in the adrenal glands. The interplay between the murine GC corticosterone and ACTH has been previously studied (van den Beukel et al., 2014). Higher GC doses, and more chronic elevation of GC inhibit BAT activity, as concluded from cell culture data and clinical observations reporting a lower ^18^FDG uptake by BAT in patients during chronic glucocorticoid therapy (Ramage et al., 2016). In rodents, GC exert inhibitory effects on BAT development and activity, most likely mediated via the glucocorticoid receptor (Soumano et al., 2000; Viengchareun et al., 2001; Armengol et al., 2012). Furthermore, in animal studies, adrenalectomy led to an increased BAT activity (Strack et al., 1995; Scarpace et al., 2000), while GC replacement normalized BAT activity (Scarpace et al., 1988, 2000). As these studies did not investigate a direct effect of ACTH on BAT activity, it remains elusive whether increased BAT activity can be attributed to the absence of GC or to increased ACTH levels as GC are strong negative regulators of ACTH secretion. Thus, the physiological relevance of ACTH in regulating BAT function may rather be indirect, depending on glucocorticoids. Notably, in obese rats the thermogenic effect of ACTH was attenuated by chronic increases in corticosterone (York and Al-Baker, 1984). However, no study so far addressed the immediate impact of GC on respiration in brown adipocytes. Therefore, we tested the thermogenic effects of ACTH and ISO after pretreatment of brown adipocytes with DEXA for up to 4 h. We found that DEXA had no effect on ACTH mediated respiration but attenuated the effect of ISO. We therefore conclude that the inhibitory action of DEXA occurs upstream of cAMP, as we observed an attenuation of the adrenergic signaling already at this level. For example, there might be an inhibition of G_s_-mediated activation of adenylyl cyclase which is selective for ADRB3 signaling. Such selectivity would require that ARDB3 and MC2R couple to different isoforms of G-proteins and/or adenylyl cyclases expressed in brown adipocytes. Two hours of DEXA treatment was sufficient to obtain a significant downregulation of the thermogenic response to ISO. This effect was more pronounced after extended DEXA treatment for 4 h. This temporal increase of inhibitory action is compatible with the activation or repression of gene transcription mediated by DEXA via the glucocorticoid receptor. DEXA treatment for 1 h had no effect, thus excluding the involvement of rapid non-genomic effects of GC.

Pertaining to systemic demands and BAT functionality, cold exposure and restraint stress, both elevating circulating ACTH levels, fundamentally differ. In the cold, metabolic fuels need to be mobilized and delivered to BAT, whereas in a fight-or-flight situation, metabolic fuels must be channeled to brain, heart and skeletal muscle. Cold exposure (24 h) led to a strong rise in ACTH plasma levels (van den Beukel et al., 2014). It remains to be clarified whether this physiological rise in ACTH levels is sufficient to transiently activate BAT thermogenesis and contribute to the initial defense of body temperature in the cold. The lipolytic activity of ACTH in WAT may also indirectly support fuel supply to BAT in the cold by augmenting the release of fatty acids into circulation. As the ACTH receptor MC2R in BAT is down-regulated in chronic cold exposure, it seems less likely, that ACTH is involved in long term stimulation and maintenance of cold-induced thermogenesis. In restraint stress, extended activation of BAT thermogenesis by ACTH and the sympathetic neurotransmitter norepinephrine would be rather counterproductive. In this context, the observed attenuation of beta-adrenergic stimulation of thermogenesis by GC avoids excessive fuel wasting in BAT and limits stress-induced hyperthermia. However, pertaining to cold stress glucocorticoid levels rise as well as norepinephrine levels (Young et al., 1982; Sesti-Costa et al., 2010). Maximal activation of BAT thermogenesis can lead to a rapid depletion of lipid stores in brown adipocytes. Acute GC-mediated downregulation of beta-adrenergic signaling might transiently hinder the sequestration of lipid stores in brown adipocytes and promote the import of metabolic fuel from circulation, for example fatty acids from lipolysis in WAT depots or triglyceride-rich lipoproteins originating from intestine and liver.

Regarding long-term effects of the HPA-axis the bidirectional effect of stress on body weight might be explained by eating behavior and BAT recruitment and thermogenesis (Razzoli et al., 2016). Obesity is associated with chronic stress and low socio-economic status and stress induced thermogenesis has been repeatedly reported in mice and humans (Lkhagvasuren et al., 2011; Kataoka et al., 2014; Robinson et al., 2016). Thus, BAT function is a determinant of the vulnerability to stress-induced obesity (Razzoli et al., 2016). An attenuation of the beta-adrenergic stimulation of BAT thermogenesis by glucocorticoids could contribute to an amplification of the obese phenotype.

In summary, we identified several activators in cell culture which can serve as potential candidates to induce BAT thermogenesis *in vivo*. As a showcase, we demonstrated that ACTH activates the canonical pathway also targeted by β-adrenergic receptor signaling for activation of BAT thermogenesis. Furthermore, we add a synthetic ACTH peptide fragment to the expanding list of thermogenic compounds. As the ACTH receptor MC2R is down-regulated in response to cold, we hypothesize that its impact on cold-induced thermogenesis is rather transient. ACTH triggers the synthesis and release of GCs from the adrenal glands which have been reported to inhibit *Ucp1* expression in brown adipocytes. Additionally, in the present study we demonstrate that the GC dexamethasone attenuates β-adrenergic receptor signaling. We conclude that stress induced GC levels *in vivo* may limit extended energy dissipation in brown adipocytes and stress-induced hyperthermia, probably in a rather transient manner. Further studies *in vivo* are required to elucidate the effects of physiological cold- and stress-induced ACTH and glucocorticoid levels on BAT thermogenesis.

## Data Availability Statement

Publicly available datasets were analyzed in this study. This data can be found here: https://www.ncbi.nlm.nih.gov/geo/query/acc.cgi?acc=GSE119452.

## Author Contributions

KS and MK conceived and designed the study. KS and JW performed the experiments. YL helped with the luciferase assay. KS analyzed the data. KS and MK wrote the manuscript. All authors read and approved the manuscript.

## Conflict of Interest Statement

The authors declare that the research was conducted in the absence of any commercial or financial relationships that could be construed as a potential conflict of interest.
